# Deletion of IQGAP1 promotes *Helicobacter pylori*-induced gastric dysplasia in mice and acquisition of cancer stem cell properties *in vitro*

**DOI:** 10.18632/oncotarget.12486

**Published:** 2016-10-06

**Authors:** Emilie Bessède, Silvia Molina, Luis Acuña Amador, Pierre Dubus, Cathy Staedel, Lucie Chambonnier, Alice Buissonnière, Elodie Sifré, Alban Giese, Lucie Bénéjat, Benoît Rousseau, Pierre Costet, David B. Sacks, Francis Mégraud, Christine Varon

**Affiliations:** ^1^Bacteriology Laboratory, University of Bordeaux, Bordeaux, France; ^2^INSERM, U853, Bordeaux, France; ^3^EA2406 Histologie et pathologie moléculaire des tumeurs, University of Bordeaux, Bordeaux, France; ^4^‘RNA: Natural and Artificial Regulation’ (ARNA) Laboratory, University of Bordeaux, Bordeaux, France; ^5^INSERM, U869, Bordeaux, France; ^6^Service Commun des Animaleries, Animalerie A2, University of Bordeaux, Bordeaux, France; ^7^Service Commun des Animaleries, Animalerie Transgénique, University of Bordeaux, Bordeaux, France; ^8^Department of Laboratory Medicine, National Institutes of Health, Bethesda, MD, USA; ^9^Experimental Pathology Platform, SIRIC BRIO, University of Bordeaux, Bordeaux, France

**Keywords:** EMT, gastric cancer, CD44, E-cadherin, Zeb

## Abstract

*Helicobacter pylori* infection is responsible for gastric carcinogenesis but host factors are also implicated. IQGAP1, a scaffolding protein of the adherens junctions interacting with E-cadherin, regulates cellular plasticity and proliferation. In mice, IQGAP1 deficiency leads to gastric hyperplasia. The aim of this study was to elucidate the consequences of IQGAP1 deletion on *H. pylori*-induced gastric carcinogenesis.

Transgenic mice deleted for *iqgap1* and WT littermates were infected with *Helicobacter* sp., and histopathological analyses of the gastric mucosa were performed. IQGAP1 and E-cadherin expression was evaluated in gastric tissues and in gastric epithelial cell lines in response to *H. pylori* infection. The consequences of IQGAP1 deletion on gastric epithelial cell behaviour and on the acquisition of cancer stem cell (CSC)-like properties were evaluated. After one year of infection, *iqgap1*+/- mice developed more preneoplastic lesions and up to 8 times more gastro-intestinal neoplasia (GIN) than WT littermates. *H. pylori* infection induced IQGAP1 and E-cadherin delocalization from cell-cell junctions. *In vitro*, knock-down of IQGAP1 favoured the acquisition of a mesenchymal phenotype and CSC-like properties induced by *H. pylori* infection.

Our results indicate that alterations in IQGAP1 signalling promote the emergence of CSCs and gastric adenocarcinoma development in the context of an *H. pylori* infection.

## INTRODUCTION

Gastric adenocarcinoma remains the third most common cause of cancer-related mortality worldwide [1]. *Helicobacter pylori* infection is involved in the carcinogenesis process and has been classified as a class 1 carcinogen by the WHO [2]. The CagA protein produced by certain *H. pylori* strains is the main pathogenic factor implicated in gastric adenocarcinoma. CagA, once injected into the host cell, disrupts the cell junctions, in particular at the level of the E-cadherin-based adherens junctions [3], and induces an epithelial to mesenchymal transition (EMT) [4] which leads to the emergence of cells with cancer stem cell (CSC) properties [5]. EMT appears to be at the origin of the adenocarcinoma initiation [6, 7]. According to Lauren's classification, 2 types of gastric carcinoma (GC) have been defined, the intestinal type and the diffuse type [8]. Recent reports on whole-genome sequencing and comprehensive molecular profiling identified new driver mutations in GC defining 4 main subtypes based on their molecular signature. Among the diffuse type GCs, in addition to well-known invalidating mutations of the E-cadherin *cdh1* gene, invalidating mutations of the *rhoA* gene were also found, in nearly 30% and 15% of the cases, respectively [9, 10]. IQ-domain GTPase-activating proteins (IQGAPs) are an evolutionary conserved family of multi-domain proteins that regulate distinct cellular processes including cell adhesion, cell migration, response to extracellular signals and cytokinesis [11, 12]. There are 3 IQGAP proteins and among them IQGAP1, the first to be described, is the only one ubiquitously expressed. IQGAP1 is located on chromosome 15q26, which corresponds to a hotspot for gene amplification, in particular in diffuse type GCs [13]. IQGAP1 is a scaffold protein interacting with numerous partners which modulates the actin cytoskeleton *via* Rac1 and Cdc42, cell/cell adhesions *via* E-cadherin, plasticity and proliferation *via* the Wnt/ß-catenin proteins, β-catenin and APC, and MEK and Erk [11]. Thus, it appears that IQGAP1, as well as E-cadherin, could play a role in gastric carcinogenesis. Different studies linked IQGAP1 expression [13-17] and localization [18] to neoplasia. The role of IQGAP1 has been studied in different models. Li *et al*. showed that aged mice lacking IQGAP1 develop gastric hyperplasia, suggesting a role for IQGAP1 in maintaining epithelial integrity during ageing [19]. Furthermore, Cai *et al*., using a murine model, showed that a *Helicobacter* sp. infection leads to a progressive shift of IQGAP1 from cytoplasmic expression to cell surface expression [20].

The above observations led to the consideration that IQGAP1 may be implicated in GC initiation and progression in the presence of *H. pylori*. Consequently our aim was to determine whether IQGAP1 inhibition favours EMT and acquisition of CSC properties in an *in vitro* model (gastric epithelial cell lines) and whether IQGAP1 inhibition accentuates the *H. pylori* carcinogenesis. Furthermore, a study of IQGAP1 deletion in mice was performed in order to better determine the potential role of IQGAP1 *in vivo* in the development of neoplastic lesions after *H. pylori* infection.

## RESULTS

### Effect of IQGAP1 deletion on the development of lesions in the gastric mucosa of mice infected by *H. pylori*

In order to determine the role of IQGAP1 in the development of lesions in the gastric mucosa in response to Helicobacter infection, infection experiments were carried out on female 129/Bl/6 mice deleted for one allele of *iqgap1* [19] and on their WT littermates. Mice were infected with either *H. felis* (an animal pathogen which is a strong inducer of gastric inflammation and pre-neoplastic lesions in mice), *H. pylori* SS1 which harbours a non-functional *cag* pathogenicity island (PAI), and *H. pylori* HPARE which harbours a functional *cag*PAI and CagA. The mice were sacrificed after 6 or 12 months for histopathological analyses as previously described [21].

Invalidation of one or two *iqgap1* alleles was described to lead to the same phenotype. In order to confirm this point, groups of *iqgap1*-/- mice, *iqgap1+/-* mice and WT littermates, were infected or not (control) with only *H. felis* to determine the consequences of invalidating one or both *iqgap1* alleles on the development of gastric inflammation and pre-neoplastic lesions 6 months post-infection (PI). We decided to work with heterozygous and not with homozygous mice in order to mimic as closely as possible the situation in patients.

**Figure 1:**
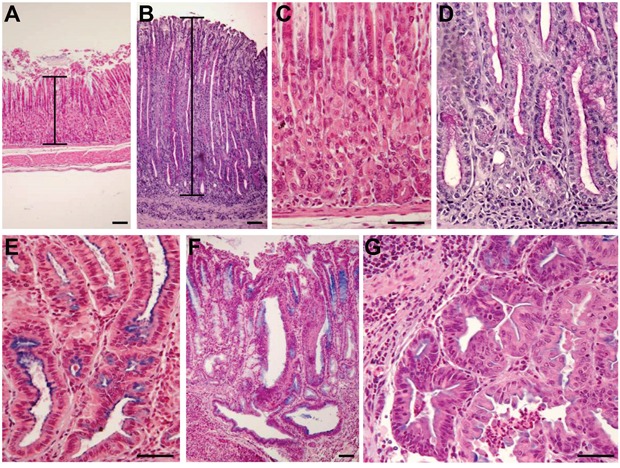
Development of histopathological lesions of the gastric mucosa after *Helicobacter pylori* infection in *iqgap1*+/- mice Representative images of histopathologic features of the gastric mucosa of uninfected **(A, C)** or *H. pylori* SS1-infected **(B, D, E, F, G)** *iqgap1*+/- mice after 12 months. (A, C) Normal gastric mucosa. (B) *H pylori*-induced hyperplasia compared to the normal thickness of the mucosa of an uninfected control mouse (A). (D) Mucinous metaplasia with mucins producing cells replacing parietal and chief cells observed in normal gastric mucosa of a control mouse (C). (E) Pseudo-intestinal metaplasia with cells resembling enterocytes and expressing mucins mainly at the apical surface. (F-G) Dysplasia with gastrointestinal intraepithelial neoplasia (GIN) penetrating the muscularis mucosa. (G) GIN with an area of less cohesive dysplastic cells. Images are representative of the lesions obtained with *Helicobacter felis* and *H. pylori* SS1 and HPARE strains in WT and *iqgap1*+/- mice. Scale bar = 50 μm.

No significant lesions were observed in uninfected mice at any time point, neither in WT nor in *iqgap1*+/- mice or *iqgap1*-/- mice (data not shown). After 6 and 12 months of infection of *iqgap1*+/- mice, both *H. pylori* strains and *H. felis* induced significant inflammation in the gastric mucosa and sub-mucosa associated with an increase in mucosal height (Figure 1), an atrophy with the replacement of parietal and chief cells by mucous-producing cells defining a mucinous metaplasia (Figure 1). Dysplastic lesions appeared 6 months PI. Their number and their severity increased overtime, reaching high-grade gastrointestinal intraepithelial neoplasia (GIN) at 12 months PI (Figure 1F-1G). At 6 months PI, no difference was observed between the WT and the *iqgap1*+/- mice (data not shown) except for GIN which developed in response to *H. felis* only in *iqgap1*+/- and to the same extent in *iqgap1*-/- mice, but not in WT mice (~40% of *H. felis*-infected *iqgap1*+/- mice and of *H. felis*-infected *iqgap1*+/- mice *vs*. 0% of *H. felis* WT mice; 8<n<10 in each group) (Table 1).

**Table 1:** Occurrence of gastrointestinal neoplasia (GIN) lesions in *iqgap1* -/-, +/- and +/+ mice after Helicobacter infection with 3 different strains

		Number of mice with GIN / total number of mice			Number of GIN lesions		
		WT	+/-	-/-	WT	+/-	-/-
**M6**	**Control**	0/9	0/10	0/8			
	***H. felis***	0/10	4/10	4/9	0	4	4
	***H. pylori SS1***	0/7	0/9		0	0	
	***H. pylori* HPARE**	0/10	0/14		0	0	
**M12**	**Control**	0/9	0/10		0	0	
	***H. felis***	12/17	17/18		29	52	
	***H. pylori SS1***	1/18	5/18		1	5	
	***H. pylori* HPARE**	0/20	3/20		0	3	

For all mice studied GIN lesions have been evaluated on 2 separated tissue section per mouse. For each group of mice (7<n<20 per experimental condition), the number of mice with GIN compared to the total number of mice in the corresponding group is indicated. The total number of GIN lesions per group corresponds to the sum of all GIN lesions recorded for all mice of the same group (some mice harbouring several GIN lesions).

**Figure 2:**
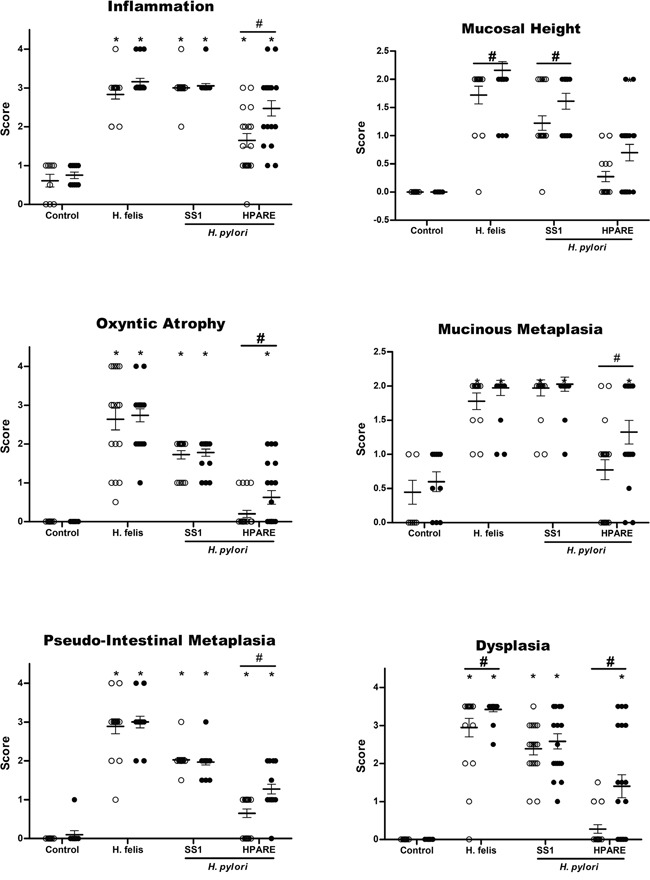
Evaluation of gastric histopathological lesions 12 months post-infection with Helicobacter Scores for gastric inflammation, mucosal height, oxyntic atrophy, mucinous metaplasia, pseudo-intestinal metaplasia, and dysplasia determined on WT (light grey) and *iqgap1*+/- (black) mice after 12 months of infection with *Helicobacter pylori* SS1, HPARE or *Helicobacter felis* (17 < n < 20) or uninfected controls (9 < n < 10) are shown for comparison. Data represent the mean ± SD. *, p < 0.05 compared to uninfected controls; #, p < 0.05 compared to WT mice.

At 12 months PI, *H. felis* induced more hyperplasia and dysplasia in *iqgap1*+/- mice compared to the WT mice (Figure 2). The non-functional *cag*PAI *H. pylori* strain SS1 only induced more hyperplasia in *iqgap1*+/- mice compared to the WT mice (Figure 2). The most important effects were observed in response to the *H. pylori* strain HPARE harbouring a functional *cag*PAI, with *iqgap1*+/- infected mice developing significantly greater inflammation, hyperplasia, atrophy, metaplasia and dysplasia (Figure 2). All of the analyzed mice were consistently positive for *H. pylori* 12 months PI as confirmed by direct culture of the bacteria and/or PCR amplification of stomach samples (data not shown). Twelve months PI with *H. felis*, almost all of the *iqgap1*+/- mice (94.5%) developed GIN lesions compared to 70.5% of their WT littermates, and the total number of GIN lesions observed in *iqgap1*+/- mice was almost twice that of WT mice (52 *vs*. 29) (Table 1). Furthermore, *iqgap1*+/- mice infected with *H. pylori* developed 8 times more GIN lesions than their WT littermates (21% *vs.* 2.6% of mice infected with *H. pylori* SS1 and HPARE).

### Expression and localization of IQGAP1 in response to *H. pylori* infection *in vivo* and in gastric cancer

**Figure 3:**
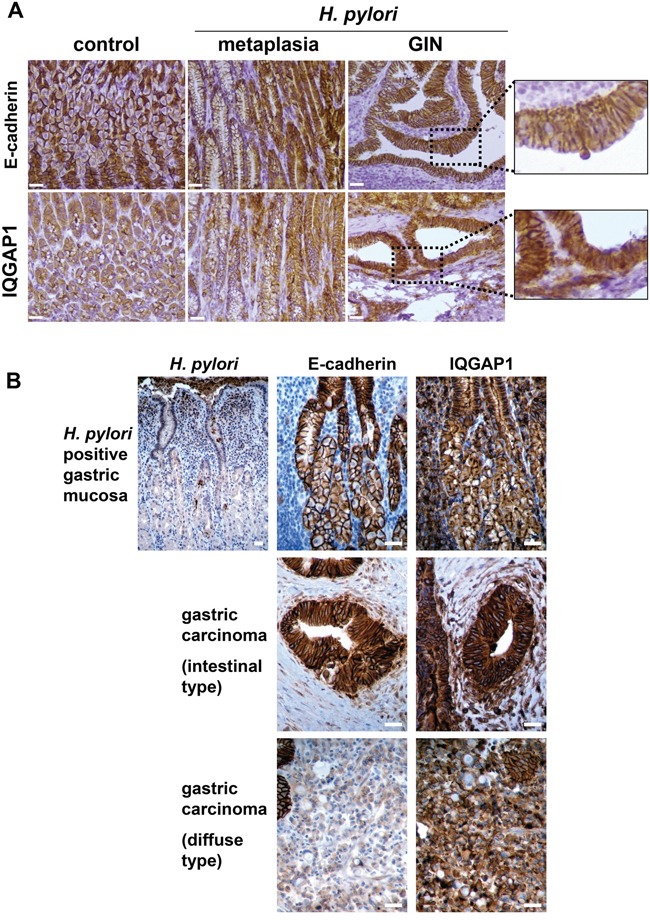
Expression of IQGAP1 and E-cadherin in gastric mucosa of mice and humans infected by *Helicobacter pylori* and in GIN and gastric carcinoma Representative images of immunohistochemistry detection (in brown) of *H. pylori*, IQGAP1, and E-cadherin on gastric PET-sections. **(A)** Gastric tissue sections from uninfected (control) or *H. pylori* SS1-infected wild type (WT) mice (12 months) having developed metaplasia of the mucinous type (left side of the metaplasia images) and/or pseudo-intestinal type (right side of the metaplasia images) and GIN. **(B)** Representative gastric tissue sections from only gastric cancer patients with *H. pylori* associated chronic gastritis (n = 6) (top row), with moderately differentiated intestinal-type adenocarcinoma (n = 6) (second row) and with diffuse type adenocarcinoma comprised of E-cadherin-negative isolated signet ring cells (n = 3) (third row). Scale bars, 25 μm.

The expression of IQGAP1 and E-cadherin was evaluated by immunohistochemistry (IHC) on the gastric mucosa of the corpus region of the stomach of WT mice 12 months PI with Helicobacter. In uninfected mice, IQGAP1 expression was detectable mainly in gastric epithelial cells compared to stromal cells and infiltrated leucocytes, and was located mainly at cell-cell junctions on the surface of parietal and chief cells, with a more intense and diffuse cytoplasmic pattern of expression in chief cells (Figure 3A). In GIN induced after *H. felis* or *H. pylori* infection, IQGAP1 remained localized at cell-cell junctions but was also detected more diffusely in the cytoplasm. E-cadherin was detected at cell-cell junctions of gastric epithelial cells in uninfected mice; its expression was lower in chief cells than in parietal or surface epithelial cells (Figure 3A). In GIN, E-cadherin localization was more diffuse but always present at cell-cell junctions.

The expression of these proteins was evaluated by IHC on tissue sections of fundic gastric mucosa from *H. pylori*-infected patients and on gastric adenocarcinoma samples (Figure 3B). IQGAP1 and E-cadherin were expressed predominantly at the epithelial cell-cell junctions in gastritis. IQGAP1 was also present in the cytoplasm but to a lesser extent. E-cadherin was poorly expressed in diffuse type GC as already reported [22], whereas in the intestinal type, E-cadherin was localized at the cell-cell junctions and to a lesser extent into the cytoplasm. IQGAP1 localization differed in GC according to the histology. In the intestinal type, IQGAP1 was expressed mainly in the cytoplasm and reduced at cell/cell junctions, whereas in the diffuse type IQGAP1 was mainly membrane related (Figure 3A).

### Effect of IQGAP1 knock-down on cancer stem cell-like properties *in vitro*

**Figure 4:**
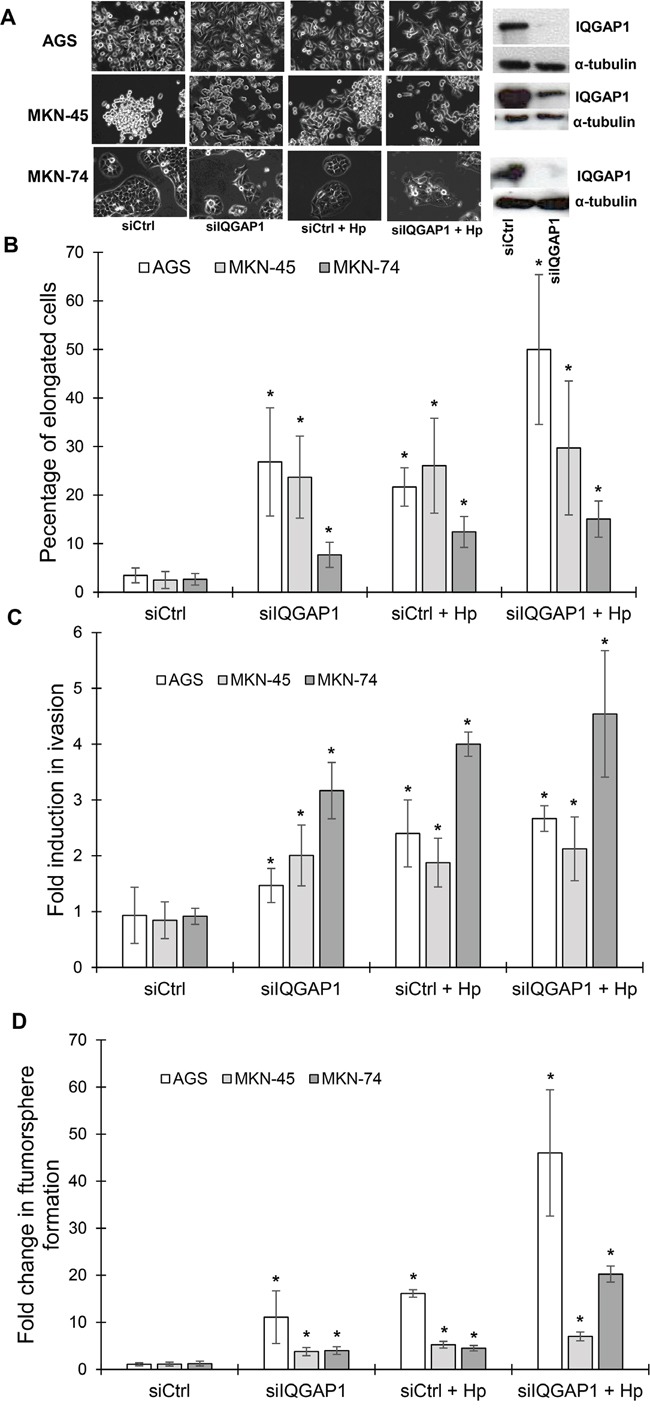
IQGAP1 inhibition mimicks *Helicobacter pylori* induced effects on gastric epithelial cell morphology, invasion capacities and tumorsphere formation AGS, MKN-45 and MKN-74 cells were transfected in a double round of transfection with the negative control (siCtrl) or IQGAP1 (siIQGAP1) siRNA, and infected or not with *H. pylori* 7.13 for 18 to 24 h. **(A)** Representative phase-contrast images of transfected cells after infection or not with *H. pylori* 7.13; and representative images of western blotting experiments showing the inhibition of expression of IQGAP1 by siIQGAP1 compared to siCtrl transfected cells. α-tubulin detection was used as a loading control of equal amounts of proteins. Scale bar: 10 μm. **(B)** Quantification of the percentage of cells harboring an elongated phenotype. **(C)** Quantification of cellular invasion after 18 h of infection in the Transwell invasion assay. * p<0.05 vs. uninfected siCtrl cells. **(D)** Quantification of spheroids obtained under non-adherent culture conditions after 5 days of infection in the tumorsphere assay.

*H. pylori* induces an EMT *in vitro* which leads to the emergence of cells expressing CD44 and possessing CSC-like properties [4-6]. As IQGAP1 is a component of adherens junctions and as these structures are destabilized following infection, the impact of IQGAP1 knock-down on *H. pylori*-promoted-EMT and CSC properties was evaluated. Therefore, gastric epithelial cells were transfected with siRNA targeting IQGAP1 (siIQGAP1) or negative control siRNA (siCtrl) and were infected or not with *H. pylori* (Figure 4). Upon IQGAP1 silencing, the percentage of cells exhibiting a “hummingbird” phenotype significantly increased from 5 to 25% in AGS and MKN-45 cells, from 5 to 10% in MKN-74 cells (Figure 4A-4B). The “hummingbird” phenotype was even more prevalent in cells infected with *H. pylori* and transfected with siIQGAP1 reaching 50%, 30% and 17% for AGS, MKN-45 and MKN-74 cells, respectively.

Some CSC-like properties of infected and/or transfected cells were studied. All of the cells transfected with siIQGAP1 were significantly more invasive (Transwell assay) than siCtrl transfected cells, indicating that IQGAP1 inhibition promotes invasion (Figure 4C). The most important effect was observed in MKN-74 cell line: transfected cells were 3 times more invasive and transfected and infected cells were 4 times more invasive than control cells. MKN-74 cells correspond to cells having intact cell-cell junctions in contrast to AGS and MKN-45 cells which have altered cell-cell junction due to the *cdh1* mutation. The same results were obtained concerning the capacity of the cells to form tumorspheres (Figure 4D). Indeed, the cells with the strongest ability to form tumorspheres were those infected and transfected with siIQGAP1, implying that the inhibition of IQGAP1 promotes tumorsphere formation in response to *H. pylori* infection, especially in AGS and MKN-74 cells where the association of the transfection and the infection led to 40 and 20 times more tumorspheres, respectively.

### Effects of *H. pylori* on the localization and expression of IQGAP1, CD44 and EMT-mediating transcription factors in gastric epithelial cells *in vitro*

**Figure 5:**
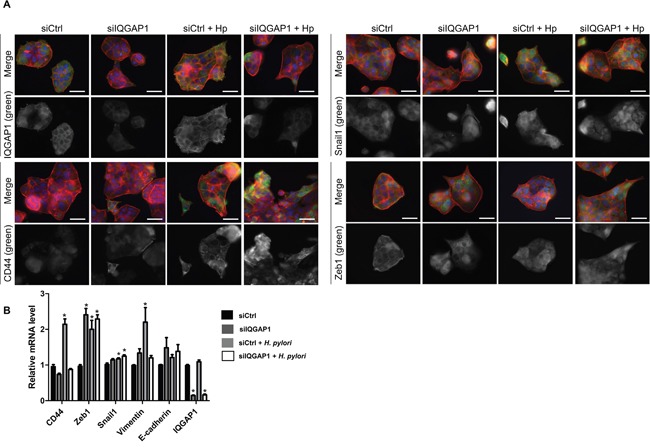
IQGAP1 inhibition accentuates *Helicobacter pylori* effect on EMT and CD44 expression in MKN-74 cells MKN74 cells were cocultured with *H. pylori* 7.13 for 24 h. **(A)** Representative images of immunofluorescent staining of IQGAP1, CD44, Snail1 and Zeb1 alone (in green) or combined with F-actin (in red, phalloidin staining) and nuclei (in blue, DAPI staining). Scale bars, 15 μm. **(B)** Relative expression levels of the mRNA encoding IQGAP1, CD44, Zeb1, Snail1, Vimentin, E-cadherin in uninfected cells, siIQGAP1-transfected cells, infected cells and infected and siIQGAP1-transfected cells by RT-qPCR (relative to HPRT1 housekeeping gene mRNA) (n=6).

**Figure 6:**
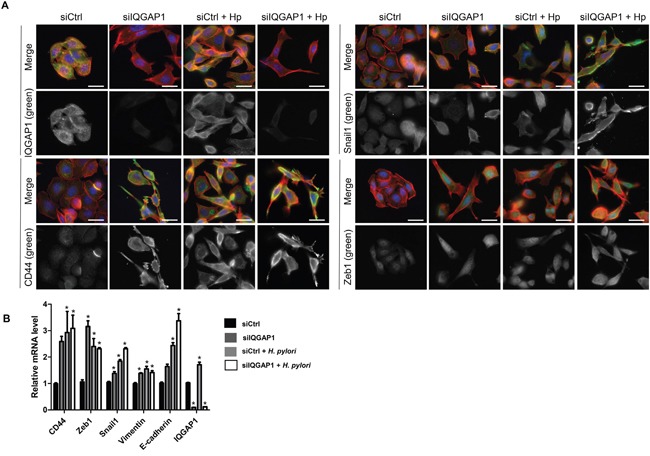
IQGAP1 inhibition accentuates *Helicobacter pylori* effect on EMT and CD44 expression in AGS cells AGS cells were cocultured with *H. pylori* 7.13 for 24 h. **(A)** Representative images of immunofluorescent staining of IQGAP1, CD44, Snail1 and Zeb1 alone (in green) or combined with F-actin (in red, phalloidin staining) and nuclei (in blue, DAPI staining). Scale bars, 10 μm. **(B)** Relative expression levels of the mRNA encoding IQGAP1, CD44, Zeb1, Snail1, Vimentin, E-cadherin in uninfected cells, siIQGAP1-transfected cells, infected cells and infected and siIQGAP1-transfected cells by RT-qPCR (relative to HPRT1 housekeeping gene mRNA) (n=6).

To study the localization of IQGAP1, CD44 and mesenchymal markers, MKN-74 gastric epithelial cells (forming cell-cell junctions) and AGS gastric epithelial cells (*cdh1* mutated, reduced cell/cell junctions) were infected with the *cag*PAI positive *H. pylori* strain 7.13 for 24 h (Figures 5 and 6). In the 2 cell lines, *H. pylori* infection induced an increase in CD44, in the EMT markers Snail1, Zeb1 and Vimentin and in IQGAP1 at the mRNA and protein levels (Figures 5 and 6). In MKN-74 cells, IQGAP1 localization changed with *H. pylori* infection: IQGAP1 was translocated from the cell-cell junctions to the cytoplasm and to membrane ruffles (Figure 5A). It is also interesting to note that inhibiting IQGAP1 and infecting the cells with *H. pylori* led to the formation of compact cell clusters with a spheroid appearance at the cell periphery and expressing high quantities of CD44 and Snail1. Furthermore, the immunofluorescence staining performed on MKN-74 confirmed that siIQGAP1 transfection favors a mesenchymal phenotype whereas the elongated cells highly express Zeb1 and to a lesser extent Snail1. The same trends were obtained with AGS cells in which IQGAP1 was mainly expressed in the cytoplasm and to a lesser extent at the cell-cell junctions and was translocated after infection to the plasma membrane (Figure 6). These results were partly confirmed *in vivo* in the mouse model, *iqgap1* +/- and -/- mice showing a higher expression of Snail and Zeb1 compared to WT mice (Supplementary Figure S1). Infection with *H. felis* significantly increased the expression of CD44 and Zeb1 in *iqgap1* +/- mice compared to uninfected WT mice, and their expression tended to be higher in *H. felis* infected *iqgap1*+/- mice than in *H. felis* infected WT mice (Supplementary Table S1).

## DISCUSSION

We previously reported that the EMT-like changes induced during *H. pylori* infection are associated with the emergence of CD44+ cells possessing CSC-like properties [5, 6]. Here, inhibition of IQGAP1 induced a similar EMT-like process due to the adherens junction destabilization and this effect was more pronounced with *H. pylori* infection.

Interestingly, we show that reducing IQGAP1 by using siRNA, led to CD44 upregulation, as well as the acquisition of invasive properties and formation of tumorspheres *in vitro*, 2 hallmarks of CSC-like cells. In addition, our *in vitro* data revealed for the first time that the association of IQGAP1 inhibition and *H. pylori* infection favors EMT and the acquisition of CSC properties. This is in agreement with different reports which considered IQGAP1 as a potential target of *H. pylori* in disrupting epithelial polarity [23]. Furthermore, the infection leads to an increase in IQGAP1 expression and also to a delocalization of IQGAP1 from cell-cell junctions and cytoplasm to the plasma membrane confirmed, using different models, the results described by Conlin *et al*. [24]. IQGAP1 knock-down led to a mesenchymal phenotype acquisition in both AGS and MKN-74 cells where IQGAP1 is not located at the same site. Indeed, in spite of the different IQGAP1 localization, observed effects are similar indicating that the effects are independent of the IQGAP1 localization.

In this study, we showed for the first time that deletion of one IQGAP1 allele in mice significantly promotes the development of high grade dysplasia in response to infection with pro-carcinogenic *H. pylori* strains. This mechanism appears to be due to a promotion and acceleration of the process of lesion development, as GIN was induced by *H. felis* after 6 months of infection in *iqgap1*+/- mice, whereas it appeared after more than 12 months in WT mice [21, 25]. Surprisingly, we were not able to reproduce the results published by Li *et al*., who observed spontaneous hyperplasia of the gastric mucosa in heterozygous and homozygous mice invalidated for *iqgap1* compared to WT littermates [19]. There were no apparent differences concerning these two independent studies, as both were performed on a 129/BL6 genetic background, with the exception of the environmental housing conditions which may differ significantly from one laboratory to another (our colony was confirmed to be negative for all pathogenic organisms including *Helicobacter* spp. before experimental infection). In the present study, aged *iqgap1*+/- mice showed no more basal inflammation or alterations of the gastric mucosa than their WT littermates, and infection with the *cag*A-positive HPARE *H. pylori* strain induced more inflammation and pre-neoplastic lesions in *iqgap1*+/- mice than in WT littermates after 12 months. Finally, *iqgap1*+/- mice developed 8 times more GIN in response to *H. pylori* infection than their WT littermates. This result suggests that the deletion of only one IQGAP1 allele could alter cell-cell junctions and favor the pro-inflammatory and pro-carcinogenic effects of *cag*A-positive *H. pylori* strains, and consequently the development of pre-neoplastic lesions in ageing animals. In humans, the carcinogenic potential of *H. pylori* is mainly associated with CagA, which can be considered to be an oncoprotein as its expression in transgenic mice is sufficient to induce carcinoma without any other stimuli [25]. These results support the hypothesis that the IQGAP1 protein is essential to maintaining the integrity of the gastric epithelium against the effects of CagA injected into host cells by carcinogenic *H. pylori* strains. Indeed, *H. pylori* and particularly the oncoprotein CagA, are known to induce a disturbance of the signalling pathways regulating the integrity of the adherens junctions and the equilibrium between cellular proliferation and the differentiation state [26-28]. Here we report that *cag*A-positive *H. pylori* strains induce a destabilization of cell/cell junctions with a delocalization of IQGAP1 and its partner E-cadherin. Little is known about the molecular events leading to the development of diffuse type gastric adenocarcinoma, as no pre-malignant precursor lesions or markers have been described. Diffuse type GCs are associated with mutations in the *cdh1* gene encoding E-cadherin in about 30% of cases. This was confirmed experimentally in one study carried out on transgenic mice in which the invalidation of one *cdh1* allele led to the development of diffuse type gastric adenocarcinoma in response to N-methyl-N-nitrosourea [22]. For the remaining 70% of the cases, other gene deregulations or mutations have been proposed. In two recent reports from the Consortium of the Human Genome Atlas and from Wang *et al.*, a molecular classification of gastric adenocarcinoma in 4 main subclasses was proposed based on their molecular profile of mutations and genetic stability [9, 10]. These two studies do not report IQGAP1 mutations among the most frequent ones described, but in 2005 Morris *et al.* reported some specific IQGAP1 mutations in a few cases of diffuse type GC [29], showing that IQGAP1 mutations are not commonly found in GC. However, our results are in agreement with those of Takemoto *et al.* concerning the localization of IQGAP1. They stated that IQGAP1 was frequently observed diffusely in the cytoplasm in intestinal type tumors whereas it was expressed at the cell membrane in diffuse type tumors [18]. In our study, the same trend was observed for neoplastic lesions in the mouse model of *H. pylori* infection and in patients with GC.

Our results prove that IQGAP1 is critical for the maintenance of normal cell physiology limiting carcinogenesis especially during *H. pylori* infection.

## MATERIALS AND METHODS

### Mice

Experiments on mice were carried out in Level 2 animal facilities at the University of Bordeaux in agreement with the local Ethics Committee (approval number 50120141-A) and in conformity with the French Committee of Genetic Engineering (approval number 4608). The *iqgap1*-null 129/BL6 were kindly provided by A. Bernards (Massachusetts General Hospital Center for Cancer Research, Charlestown, MA, USA) [19]. Heterozygous *iqgap1*+/- 129/BL6 males were bred with wild type (WT) C57Bl/6 females, and heterozygous *iqgap1*+/- females and WT littermates were generated. Groups of WT and *iqgap1*+/- mice (7 < n < 17 for each group) were force-fed with *H. pylori* SS1 *H. pylori* HPARE, *Helicobacter felis* (both kindly provided by A. Labigne, Pasteur Institute, Paris, France) or phosphate buffered saline (PBS) for control mice, at 6 weeks of age with 200 μl of 10.8 CFU/ml of *Helicobacter* sp. suspension every other day for a total of three doses [21]. A group of *iqgap1*-/- mice was challenged with *Helicobacter felis* (n = 9) or PBS (n = 8) for 6 months. After 6 or 12 months of infection, mice were euthanized by cervical dislocation; stomachs were collected aseptically, opened along the greater curvature, washed in PBS and sectioned longitudinally into 2 pieces along the smaller curvature from the squamocolumnar junction through the pylorus. The first part of the stomach was fixed for 24 h in 3.7% neutral-buffered formalin (Sigma, Saint-Quentin Fallavier, France), followed by standard histological processing and paraffin embedding. The second part was homogenized in 200 μL of brucella broth to confirm *H. pylori* colonization by bacterial culture and PCR as previously described [21].

Information on protocols used for bacterial culture, tissue processing, histopathological scoring, statistical analysis, cell culture experiments, and qRT-PCR is provided in the Supplementary Materials and Methods. AGS, MKN-45 and MKN-74 cell lines were used for the *in vitro* part. AGS cells are known to be predisposed to the EMT process since they have a *cdh1* mutation. Thus, they are a good model to study the *H. pylori* transforming effect. AGS cells were used to study the role of IQGAP1 independently of its junctional role.

## SUPPLEMENTARY FIGURES AND TABLES



## References

[1] Ferlay J, Soerjomataram I, Dikshit R, Eser S, Mathers C, Rebelo M, Parkin DM, Forman D, Bray F (2014). Cancer incidence and mortality worldwide: sources, methods and major patterns in GLOBOCAN 2012. International journal of cancer.

[2] Schistosomes,liver flukes and Helicobacter pylori (1994). IARC Working Group on the Evaluation of Carcinogenic Risks to Humans. Lyon.

[3] Amieva MR, Vogelmann R, Covacci A, Tompkins LS, Nelson WJ, Falkow S (2003). Disruption of the epithelial apical-junctional complex by *Helicobacter pylori* CagA. Science.

[4] Baud J, Varon C, Chabas S, Chambonnier L, Darfeuille F, Staedel C (2013). *Helicobacter pylori* initiates a mesenchymal transition through ZEB1 in gastric epithelial cells. PloS one.

[5] Bessède E, Staedel C, Acuna Amador LA, Nguyen PH, Chambonnier L, Hatakeyama M, Belleannee G, Mégraud F, Varon C (2013). *Helicobacter pylori* generates cells with cancer stem cell properties via epithelial-mesenchymal transition-like changes. Oncogene.

[6] Bessède E, Dubus P, Mégraud F, Varon C (2015). *Helicobacter pylori* infection and stem cells at the origin of gastric cancer. Oncogene.

[7] Mani SA, Guo W, Liao MJ, Eaton EN, Ayyanan A, Zhou AY, Brooks M, Reinhard F, Zhang CC, Shipitsin M, Campbell LL, Polyak K (2008). The epithelial-mesenchymal transition generates cells with properties of stem cells. Cell.

[8] Lauren P (1965). The two histological main types of gastric carcinoma: diffuse and so-called intestinal-type carcinoma an attempt at a histo-clinical classification. Acta Pathol et Microbiol Scand.

[9] Network TCGAR (2014). Comprehensive molecular characterization of gastric adenocarcinoma. Nature.

[10] Wang K, Yuen ST, Xu J, Lee SP, Yan HH, Shi ST, Siu HC, Deng S, Chu KM, Law S, Chan KH, Chan AS (2014). Whole-genome sequencing and comprehensive molecular profiling identify new driver mutations in gastric cancer. Nature Gen.

[11] Brown MD, Sacks DB (2006). IQGAP1 in cellular signaling: bridging the GAP. Trends Cell Biol.

[12] Noritake J, Watanabe T, Sato K, Wang S, Kaibuchi K (2005). IQGAP1: a key regulator of adhesion and migration. J Cell Sci.

[13] Sugimoto N, Imoto I, Fukuda Y, Kurihara N, Kuroda S, Tanigami A, Kaibuchi K, Kamiyama R, Inazawa J (2001). IQGAP1, a negative regulator of cell-cell adhesion, is upregulated by gene amplification at 15q26 in gastric cancer cell lines HSC39 and 40A. J Human Gen.

[14] Briggs MW, Sacks DB (2003). IQGAP proteins are integral components of cytoskeletal regulation. EMBO Rep.

[15] Jadeski L, Mataraza JM, Jeong HW, Li Z, Sacks DB (2008). IQGAP1 stimulates proliferation and enhances tumorigenesis of human breast epithelial cells. J Biol Chem.

[16] Nabeshima K, Shimao Y, Inoue T, Koono M (2002). Immunohistochemical analysis of IQGAP1 expression in human colorectal carcinomas: its overexpression in carcinomas and association with invasion fronts. Cancer Lett.

[17] Walch A, Seidl S, Hermannstadter C, Rauser S, Deplazes J, Langer R, von Weyhern CH, Sarbia M, Busch R, Feith M, Gillen S, Hofler H (2008). Combined analysis of Rac1, IQGAP1, Tiam1 and E-cadherin expression in gastric cancer. Mod Pathol.

[18] Takemoto H, Doki Y, Shiozaki H, Imamura H, Utsunomiya T, Miyata H, Yano M, Inoue M, Fujiwara Y, Monden M (2001). Localization of IQGAP1 is inversely correlated with intercellular adhesion mediated by e-cadherin in gastric cancers. Int JCancer.

[19] Li S, Wang Q, Chakladar A, Bronson RT, Bernards A (2000). Gastric hyperplasia in mice lacking the putative Cdc42 effector IQGAP1. Mol Cell Biol.

[20] Cai X, Carlson J, Stoicov C, Li H, Wang TC, Houghton J (2005). Helicobacter felis eradication restores normal architecture and inhibits gastric cancer progression in C57BL/6 mice. Gastroenterology.

[21] Varon C, Dubus P, Mazurier F, Asencio C, Chambonnier L, Ferrand J, Giese A, Senant-Dugot N, Carlotti M, Mégraud F (2012). *Helicobacter pylori* infection recruits bone marrow-derived cells that participate in gastric preneoplasia in mice. Gastroenterology.

[22] Humar B, Blair V, Charlton A, More H, Martin I, Guilford P (2009). E-cadherin deficiency initiates gastric signet-ring cell carcinoma in mice and man. Cancer Res.

[23] Osman MA, Bloom GS, Tagoe EA (2013). *Helicobacter pylori*-induced alteration of epithelial cell signaling and polarity: a possible mechanism of gastric carcinoma etiology and disparity. Cytoskeleton.

[24] Conlin VS, Curtis SB, Zhao Y, Moore ED, Smith VC, Meloche RM, Finlay BB, Buchan AM (2004). *Helicobacter pylori* infection targets adherens junction regulatory proteins and results in increased rates of migration in human gastric epithelial cells. Infect Imm.

[25] Ohnishi N, Yuasa H, Tanaka S, Sawa H, Miura M, Matsui A, Higashi H, Musashi M, Iwabuchi K, Suzuki M, Yamada G, Azuma T (2008). Transgenic expression of *Helicobacter pylori* CagA induces gastrointestinal and hematopoietic neoplasms in mouse. Proc Nat Acad Scie U SA.

[26] Franco AT, Johnston E, Krishna U, Yamaoka Y, Israel DA, Nagy TA, Wroblewski LE, Piazuelo MB, Correa P, Peek RM (2008). Regulation of gastric carcinogenesis by *Helicobacter pylori* virulence factors. Cancer Res.

[27] Murata-Kamiya N, Kurashima Y, Teishikata Y, Yamahashi Y, Saito Y, Higashi H, Aburatani H, Akiyama T, Peek RM, Azuma T, Hatakeyama M (2007). *Helicobacter pylori* CagA interacts with E-cadherin and deregulates the beta-catenin signal that promotes intestinal transdifferentiation in gastric epithelial cells. Oncogene.

[28] Saito Y, Murata-Kamiya N, Hirayama T, Ohba Y, Hatakeyama M (2010). Conversion of *Helicobacter pylori* CagA from senescence inducer to oncogenic driver through polarity-dependent regulation of p21. J Ex Med.

[29] Morris LE, Bloom GS, Frierson HF, Powell SM (2005). Nucleotide variants within the IQGAP1 gene in diffuse-type gastric cancers. Genes, chromosomes & cancer.

