# Supplementary material for: Deletion of IQGAP1 promotes Helicobacter pylori-induced gastric dysplasia in mice and acquisition of cancer stem cell properties in vitro
Source: Oncotarget. 2016 Oct 6;7(49):80688–99. doi: 10.18632/oncotarget.12486 (PMC5340252; doi:10.18632/oncotarget.12486)
Supplement: Supplementary file 1 [file oncotarget-07-80688-s001.pdf]

Deletion of IQGAP1 promotes *Helicobacter pylori*-induced gastric dysplasia in mice and acquisition of cancer stem cell properties *in vitro*

SUPPLEMENTARY MATERIALS AND METHODS

Human gastric tissues

Gastric tissue samples from consenting patients (male and female, 68–85 years old) undergoing gastrectomy for distant non-cardia gastric adenocarcinoma were included in the study, in agreement with the tumor bank of the Bordeaux University Hospital Center (France). Only gastric cancer patients were included. Tumors (moderately differentiated intestinal-type adenocarcinoma and diffuse type adenocarcinoma with signet ring cells according to Lauren's classification criteria) and healthy gastric mucosa distant from the tumor site and presenting a non-atrophic gastritis associated with a positive *H. pylori* detection were studied, as previously described [4].

Bacterial culture

The mouse adapted *H. pylori* strains SS1 and HPARE and *H. felis* strain ATTC 49179 were used for mice experiments. *H. pylori* strain 7.13 was kindly provided by R. Peek (Vanderbilt University, Nashville, TN, USA). All strains were cultured as previously described [4, 12]. For coculture, *H. pylori* strains were grown at 37°C for 24 h, resuspended in PBS and adjusted to an $OD_{600\ nm} = 1$ (corresponding to $2x10^8$ CFU/ml) in PBS before infection. The homogenates from gastric tissues were seeded on selective blood agar media and incubated under microaerobic conditions at 37°C for 3 to 5 days for the culture of *H. pylori.* The Helicobacter positive status was confirmed by PCR amplification of specific genes as previously described [12].

Epithelial cell culture

Human gastric adenocarcinoma epithelial cell lines AGS, MKN74 and MKN-45 were cultured in DMEM - nutrient mixture F12 for AGS, and RPMI 1640 for MKN (both from Invitrogen, Cergy-Pontoise, France), both supplemented with 10% heat-inactivated fetal bovine serum (FBS), and vancomycin at 50 µg/ml (Sigma) in a humidified 5% CO_2 atmosphere at 37°C as previously described [4].

Immunofluorescent stainings

MKN-74 or AGS cells cultured on glass coverslips (25,000 cells/well) were fixed with 4% paraformaldehyde in PBS for 10 min and processed for immunofluorescent staining as previously described [4, 13]. Primary and secondary antibodies were used at the following concentrations: 1:100 for rabbit anti-IQGAP1 antibodies (Santa Cruz Biotechnology, Santa Cruz, CA, USA), 1:100 for mouse anti-E-cadherin (BD Bioscience, Le Pont de Claix, France), 1:100 for rabbit anti-Zeb1 (Bethyl, Montgomery, TX 77356 USA), 1:100 for mouse anti-CD44 (BD Bioscience), and 1:300 for anti-mouse or anti-rabbit Alexa-488-labelled secondary antibodies. Alexa-546-labelled phalloidin (1:300) and DAPI (1:100) were used for F-actin and nuclear staining, respectively (all from Molecular Probes, Invitrogen, Cergy-Pontoise, France). For fluorescence imaging, cells were analyzed using an Eclipse 50*i* epi-fluorescence microscope (Nikon, Champigny sur Marne, France) equipped with Nis Element acquisition software and a x40 (numerical aperture, 1.3) oil immersion objective.

Histology and immunohistochemistry

Gastric tissue samples from gastrectomies for GC were studied, in agreement with the tumor bank at the Bordeaux University Hospital and the Bergonié Institute (Bordeaux, France). Paired healthy gastric mucosa and tumors were analyzed by immunohistochemistry (IHC) on 3.7% neutral-buffered, formalin-fixed and paraffin-embedded tissue (PET). Three µm thick sections from human or mouse PET were processed for hematoxylin/eosin/safran (HES) staining, periodic acid schiff (PAS) counterstained with hematoxylin (all from Labonord, Templemars, France) or alcian blue (AB) counterstained with nuclear fast red (both from Sigma) following standard protocols [12]. Standard IHC protocols were used with rabbit anti-*H. pylori* (1:100, 1 h), rabbit anti-IQGAP1 (1:300, 2 h), mouse anti-E-cadherin (1:200, 1 h), mouse anti-CD44 (1:100, 2 h; Pharmingen), rabbit anti-ZEB1 (1:100, 2 h) or rabbit anti-Snail (1:100, 1 h; Santa Cruz Biotechnology) antibodies as previously described [4, 12]. Anti-mouse and anti-rabbit Envision systems and the DAB revelation system (all from DAKO, Courtaboeuf,

France) were used. Counterstainings were performed with hemalun.

Quantification scoring and statistical analysis

Scoring criteria for quantification of inflammation, mucosal height, oxyntic atrophy and dysplasia were determined as previously described [12]. Relative quantification of the expression of CD44 in the gastric epithelium was determined in a blind lecture using a scale from 0 to 4 as described previsouly [4] with the following criteria: 0: no staining, 1: <5% of positive epithelial cells; 2: 5 to <25% of positive cells; 3: 25 to <50% of positive cells; 4: ≥50% of positive epithelial cells. For Zeb1 which is discretely diffusely expressed in most of epithelial cells, scores with the same scale were determined but for the quantification of cells with a strong nuclear staining. For Snail, which is detected in almost all epithelial cells, scores with the same scale were determined but for the quantification of cells with a strong staining.

Invasion assay

After cell recovery, 50,000 cells per condition were placed in the upper side of a 8 µm pore size Transwell insert in 24-well culture plates with medium containing 5% FBS. For invasion assays, inserts were previously coated with 0.05 mg/mL of rat type I collagen (BD Biosciences) for 40 min at 37°C. After 18 h of incubation with cells at 37°C, the Transwell inserts were fixed in cold methanol and processed for HES staining, as previously described [4]. Cells having migrated through the lower side of the inserts were counted on five different randomly chosen fields per insert under light microscopy using a x 20 objective.

Tumorsphere assay

After cell recovery, 500 cells were plated on non-adherent 96-well culture plates (coated with 10% polyHEMA (Sigma) solution in absolute ethanol and dried overnight at 56°C). After plating, cells were incubated for 5 days at 37°C in a serum-free medium consisting of DMEM-F12 Glutamax supplemented with 20 ng/ml of EGF, 10 ng/ml of basic-FGF, 1:100 N2-supplement 100X, 0.3% glucose, and 50 µg/ml of vancomycin (all from Invitrogen and Sigma). The number of spheroids (tumorspheres) per well was counted under light microscopy using a x 20 objective.

siRNA transfection and western blotting

Transfection of small interfering RNA (siRNA) and western blotting experiments were performed as previously described [4, 14]. Two rounds of transfection into AGS, MKN-45 or MKN74 cells were performed using Lipofectamin 2000 (Life Technologies) as transfection agent as recommended. Non-silencing siRNA (siCtrl) with no known homology to mammalian genes was used as negative control (from Dharmacon, Lafayette, CO, USA), and siRNA directed against human IQGAP1 (siIQGAP1, 5′-UGAAGCUAUUGACCGUAGAdTdT-3′) kindly provided by P. Auguste (INSERM U1029, University of Bordeaux, Bordeaux, France) were used. Western blotting experiments on cell lysates collected after 48 h of transfection were performed as previously described [4, 14] using rabbit anti-IQGAP1 antibodies (1:2,000) and mouse anti-α-tubulin (1:10,000, Sigma). Proteins were detected by chemiluminescence (ECL+, Amersham Pharmacia Biotech, Piscataway, NJ, USA) using horseradish peroxidase-coupled anti-mouse secondary antibodies (DAKO).

RNA extraction and quantitative RT-PCR

Total cellular RNAs were extracted using Trizol reagent (Invitrogen) and quantified by their absorbance at 260 nm. Retrotranscription was performed with 1 µg of total RNAs using the Quantitect Reverse Transcription (RT) kit (Qiagen), according to the manufacturer's recommendations. Quantitative PCR was performed using specific primers at 0.3 µM and Maxima SYBR Green qPCR master mix (Fermentas, Courtabœuf, France).

Statistical analysis

Quantification values represent the means of three or more independent experiments, each performed in triplicate or more ± standard deviation (SD). Statistical analyses were performed using the non-parametric Kruskal-Wallis, for mice scoring results, and Mann Whitney tests on SPSS16.0F software (SPSS Inc., Chicago, IL, USA).

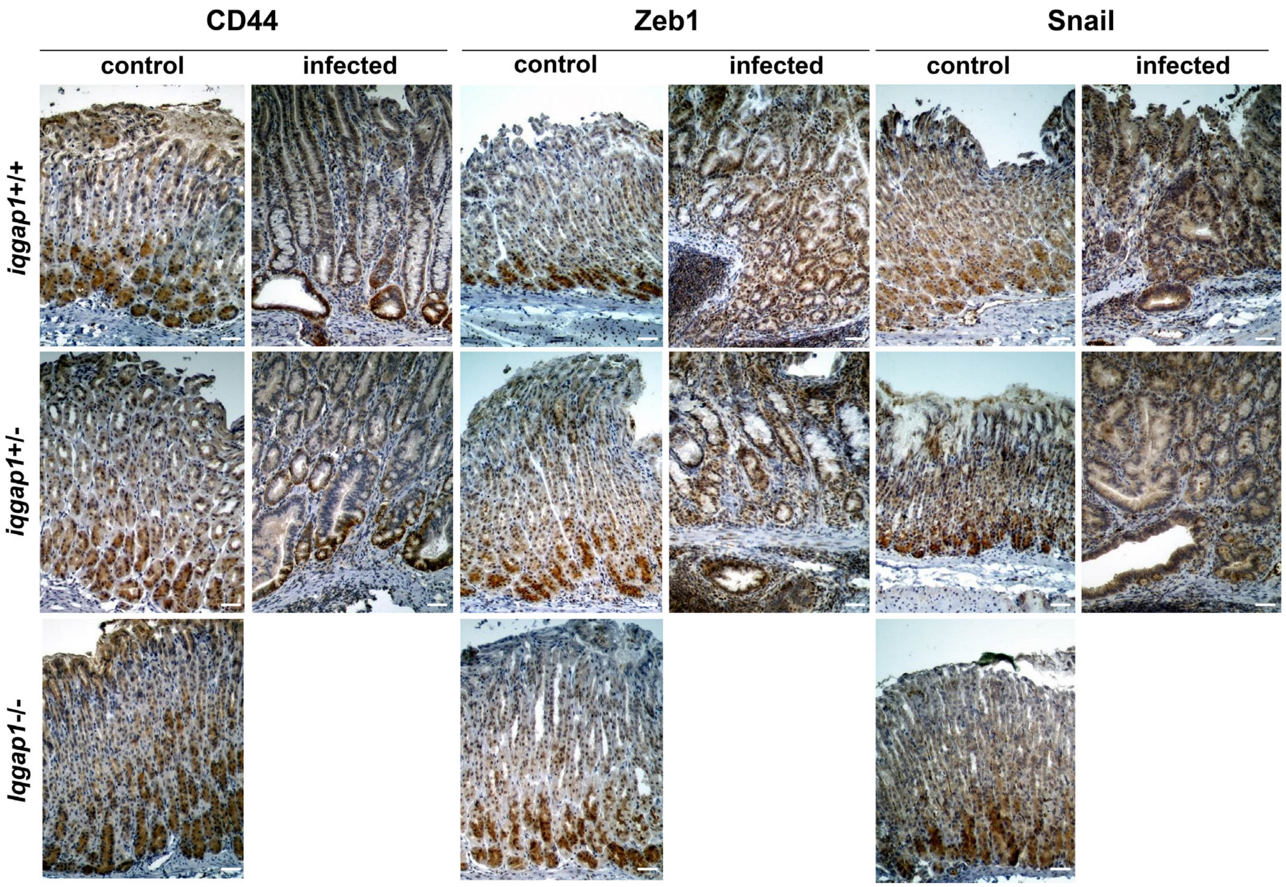

Supplementary Figure S1: Representative images of immunohistochemistry detection (in brown) of CD44, Zeb1 and Snail on mouse gastric PET-sections. Gastric tissue sections from uninfected (control) or *H. felis*-infected wild type (WT), *iqgap1*+/- and *iqgap1*-/- mice (12 months) having developed metaplasia and GIN. (4<n<5). Scale bars, 50 µm.

Supplementary Table S1: Relative expression in gastric epithelial glands of CD44, ZEB1 and Snail determined by scoring analyses of immunohistochemistry stainings of *iqgap*1$^{+/+}$, *iqgap*1$^{+/-}$ and *iqgap*1$^{-/-}$ mice after one year of infection or not with *H. felis* (4<n<5 mice per condition)

	Relative expression in gastric epithelial glands (mean ± SD; significance *vs.* uninfected *iqgap*1$^{+/+}$ mice)		
	**CD44**	**ZEB1**	**Snail**
**Uninfected *iqgap*1$^{+/+}$ mice (n = 4)**	0,5 ± 0,57	1,125 ± 0,75	1,25 ± 0,50
**Uninfected *iqgap*1$^{+/-}$ mice (n= 5)**	0,75 ± 0,50 NS	1,75 ± 0,50 NS	2,00 ± 0,00 p<0,05
**uninfected *iqgap*1$^{-/-}$ mice (n= 4)**	0,75 ± 0,50 NS	2,00 ± 0,00 p<0,05	1,50 ± 0,57 NS
***H. felis* infected *iqgap*1$^{+/+}$ mice (n= 4)**	1,50 ± 0,57 p<0,05	1,25 ± 0,64 NS	2,00 ± 0,81 NS
***H. felis* infected *iqgap*1$^{+/-}$ mice (n= 5)**	2,80 ± 0,84 p<0,01	2,25 ± 0,50 p<0,05	2,50 ± 0,57 NS

NS, not significant.
